# A comprehensive analysis of China’s plum industry: production dynamics, trade imbalances, technological innovation, and future directions

**DOI:** 10.3389/fpls.2026.1874469

**Published:** 2026-08-21

**Authors:** Yicen Xu, Zhixiang Zhang, Haijuan Zhao, Bijun Wang, Yi Hao, Shaoyi Sui, Yuping Zhang, Ming Xu, Yujun Zhang, Xiaoxue Ma, Jiacheng Liu, Jia Liu, Bo Fang, Hong Chen, Yanbo Zhang, Chaoyang Liu, Juanjuan Wu, Hailong Sun, Tanna Li, Weisheng Liu, Shuo Liu

**Affiliations:** 1 Liaoning Institute of Pomology, Yingkou, China; 2 State Key Laboratory for Biology of Plant Diseases and Insect Pests, Institute of Plant Protection, Chinese Academy of Agricultural Sciences, Beijing, China; 3 Horticulture Research Institute, Sichuan Academy of Agricultural Sciences, Chengdu, China; 4 Chongqing Academy of Agricultural Sciences, Chongqing, China; 5 College of Agriculture, Guizhou University, Guiyang, China; 6 Jilin Academy of Agricultural Sciences (Northeast Agricultural Research Center of China), Changchun, China; 7 Key Laboratory of Biology and Germplasm Enhancement of Horticultural Crops in South China, Ministry of Agriculture and Rural Affairs, College of Horticulture, South China Agricultural University, Guangzhou, China; 8 Institute of Horticultural Research, Hunan Academy of Agricultural Sciences, Changsha, China; 9 Institute of Pomology of Chinese Academy of Agricultural Science, Key Laboratory of Horticultural Crops Germplasm Resources Utilization, Ministry of Agriculture and Rural Affairs, Xingcheng, China; 10 Shenyang Agriculture University, Shenyang, China

**Keywords:** China agricultural modernization, horticultural policy, plum industry, production dynamics, *Prunus salicina*, rural revitalization, technological bottleneck, trade asymmetry

## Abstract

One of the traditional “Five Fruits” (五果) of China with origins traceable to the Huangdi Neijing (〚黄帝内经〛, c. 300 BCE), the plum (*Prunus salicina* Lindl.) constitutes both a cultural heritage crop and a strategically important horticultural commodity for rural revitalization and agricultural value-chain upgrading. This review systematically analyzes China’s plum industry across four analytical dimensions—production dynamics, trade asymmetries, technological innovation, and strategic future directions—covering the 2021–2023 period, for which FAOSTAT reports complete validated statistics. Key quantitative findings include: China’s 2023 cultivation area of 2,009 kha (>75% of global total), production of 6.89 Mt (55.3% of global output), a trade deficit expanding from USD 86 million (2018) to USD 267 million (2022), and import unit-prices averaging twice export unit-prices, with Chile alone supplying 84.45% of China’s fresh plum imports. The review identifies five structural determinants of this import-export asymmetry (varietal quality mismatch, consumer preference divergence, postharvest infrastructure deficit, market segmentation, and international competitiveness gaps) and proposes a Technology Deployment Index revealing a severe gap between available and deployed technologies, particularly for biological control (<10% adoption despite >70% availability). A three-tier strategic roadmap (short-term 1–5 years, medium-term 5–10 years, long-term 10+ years) integrates breeding, cultivation, plant protection, processing, marketing, and policy priorities. This work fills a critical gap in the literature: whereas prior reviews have focused on specific aspects (phytochemicals, breeding, or postharvest physiology in isolation), no comprehensive analytical synthesis of the entire industrial value chain with explicit quantitative benchmarking and a structured strategic framework has been available for China’s plum sector. The review provides actionable intelligence for policymakers, researchers, and industry stakeholders pursuing quality-driven industrial transformation under the Big Food Concept (大食物观) strategic framework.

## Graphical Abstract

**Figure:**
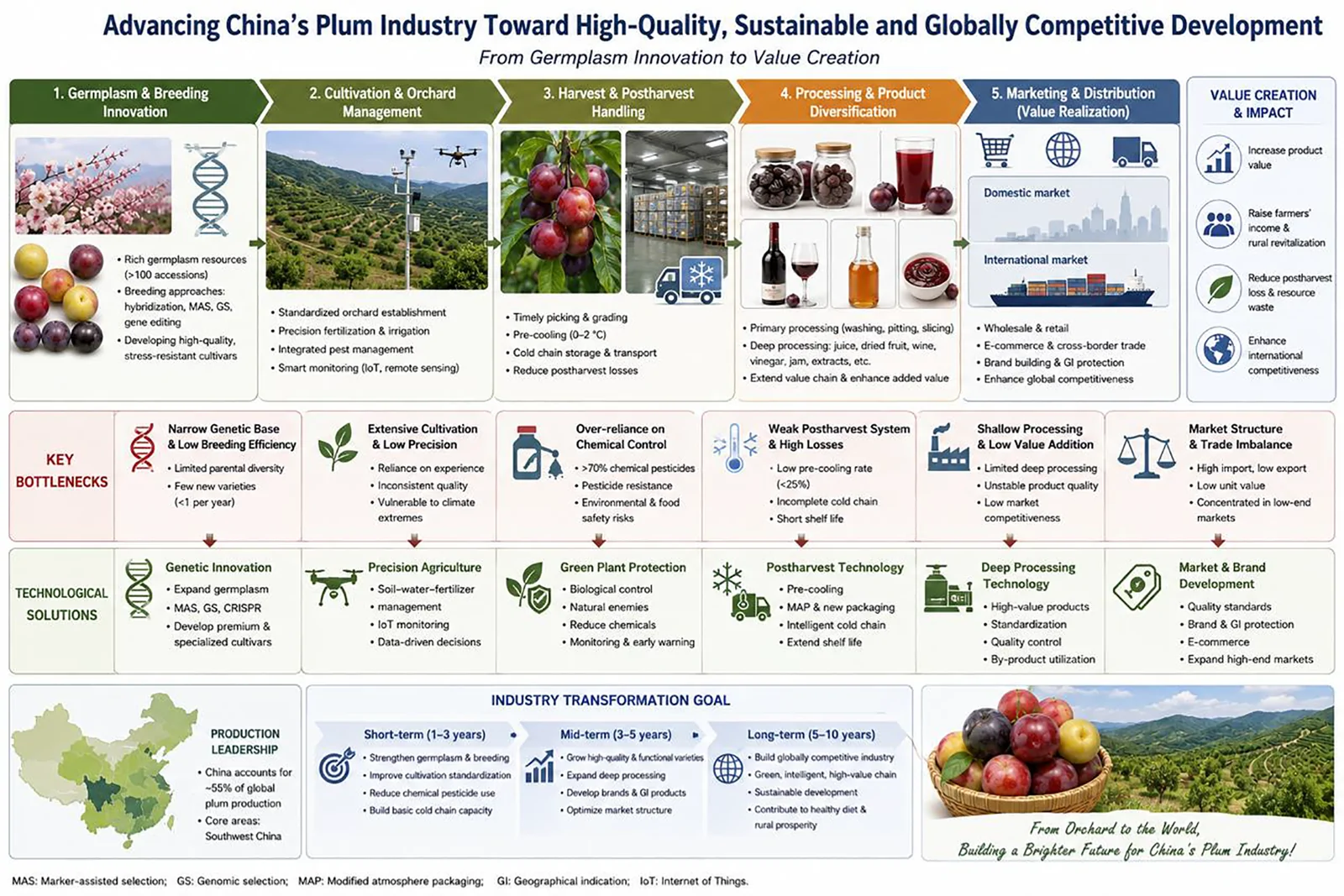
This integrated roadmap systematically frames the full industrial chain of Chinese plum production covering five sequential segments: germplasm and breeding innovation, standardized cultivation management, harvest and postharvest handling, diversified deep processing, and brand marketing and distribution. For each upstream-to-downstream link, the image first summarizes current development status and core functions, then identifies corresponding key industrial bottlenecks including narrow genetic basis, low precision cultivation, overuse of chemical pesticides, incomplete cold-chain infrastructure, shallow processing, and unbalanced market trade. Targeted technological solutions are matched to each bottleneck, covering genomic breeding, smart precision agriculture, green pest control, advanced postharvest logistics, high-value deep processing, and brand & geographical indication development. Finally, the image sets phased transformation goals divided into short-term (1–3 years), mid-term (3–5 years) and long-term (5–10 years), anchored on China’s dominant position in global plum output, to guide the industry toward sustainable, high-value and internationally competitive development from germplasm innovation to full value realization.

## 1 Introduction

The Chinese plum (*Prunus salicina* Lindl.), native to the Yangtze River watershed of central-southern China, is a deciduous fruit crop with a documented cultivation history exceeding 2,500 years, archaeologically attested in texts such as the Qimin Yaoshu (《齐民要术》, 544 CE) and the Erya (《尔雅》, c. 3rd century BCE). Together with peach (*Prunus persica*), apricot (*Prunus armeniaca*), chestnut (*Castanea mollissima*), and jujube (*Ziziphus jujuba*), plum constitutes one of the traditional “Five Fruits” (五果) classified in the Huangdi Neijing’s Yellow Emperor’s Classic of Internal Medicine (《黄帝内经·素问》), a doctrine that underpinned Chinese agricultural botany for over two millennia. Currently, *P. salicina* is the most widely cultivated *Prunus* species globally in terms of harvested area, with China alone accounting for approximately 75% of the world’s cultivation area and 55% of global output (FAO, 2023). Against the backdrop of new shifts in agricultural development paradigms and evolving demands for science and technology, the *P. salicina* industry—an integral component of characteristic and regional agriculture—confronts a dual landscape of opportunities and challenges (Wu, 2022). The implementation of the Big Food Concept (大食物观), formally elevated by chairman in 2022 as a strategic framework for expanding food supply channels beyond staple grains to encompass horticultural commodities, and the advancement of the local specialty products development initiative have emerged as core tasks driving the sustainable growth of this industry (China Policy Team, 2025; USDA GAIN, 2024).

Despite the agronomic and economic significance of *P. salicina*, no comprehensive analytical review has hitherto synthesized the entire industrial value chain—from production statistics and trade patterns through technological bottlenecks to strategic future directions—within a single quantitative framework. Prior reviews have addressed specific domains in isolation: Igwe and Charlton (2016) conducted a systematic review of health effects restricted to *Prunus domestica* and *P. salicina* phytochemicals and bioactivity; Sottile et al. (2022) focused narrowly on genetic potential and breeding constraints in European plum programs; Xia et al. (2023) examined phenotypic variation in external and internal fruit quality without industrial context; and Ricardo-Rodrigues et al. (2023) reviewed postharvest quality parameters for major *Prunus* species without production-trade integration. This review fills that gap by integrating production economics, international trade analysis, technological bottleneck assessment, and strategic roadmap development into a unified framework. Specifically, this review aims to: (i) provide the first comprehensive quantitative synthesis of China’s plum industry across the full value chain during 2021–2023; (ii) identify the structural determinants underlying China’s “high import, low export” trade paradox through multi-dimensional causal analysis; (iii) evaluate technological deployment gaps across breeding, cultivation, plant protection, storage, and processing domains using a novel Technology Deployment Index; and (iv) propose a time-phased strategic roadmap integrating short-, medium-, and long-term priorities with explicit quantitative targets. By integrating findings from recent empirical studies and technical reports, this work seeks to provide both theoretical support and practical guidance for promoting technological innovation, *e.g*., precision cultivation techniques, genetic improvement of elite varieties and industrial upgrading, *e.g*., extension of the value chain, standardization of production systems in the global plum sector (Sottile et al., 2022).

### 1.1 Review methodology: PRISMA-ScR framework

This review was conducted following the Preferred Reporting Items for Systematic reviews and Meta-Analyses Extension for Scoping Reviews (PRISMA-ScR) framework (Tricco et al., 2018; Page et al., 2021). PRISMA-ScR was selected because this study aims to map the extent, range, and nature of research activity across the entire Chinese plum industrial value chain, rather than to synthesize quantitative effect estimates.

Databases and data sources. Five primary databases were queried: (i) Web of Science Core Collection (2015–2026); (ii) Scopus (2015–2026); (iii) PubMed/MEDLINE (2015–2026); (iv) CNKI (2015–2026); and (v) FAOSTAT (1980–2023). Grey literature was collected from USDA GAIN Reports, Central No. 1 Documents, “14th Five-Year Plan” agricultural sectors, and industry association publications.

Search strategy and keywords. The search strategy combined three categories using Boolean operators: (1) crop terms: “plum” OR “Prunus salicina” OR “Prunus domestica” OR “李”; (2) thematic terms: “production dynamics” OR “trade imbalance” OR “technological innovation” OR “value chain” OR “breeding” OR “postharvest” OR “processing” OR “rural revitalization”; (3) geographic terms: “China” OR “Chinese”.

Inclusion and exclusion criteria. Studies were eligible if: (i) published in peer-reviewed journals (2015–2026), grey literature, or official statistics; (ii) contained empirical data relevant to plum production, breeding, trade, postharvest technology, or processing; (iii) focused on Prunus species with commercial cultivation in China. Studies were excluded if: (i) non-English/non-Chinese (unless English abstract available); (ii) opinion pieces without empirical content; (iii) duplicated included study; or (iv) lacked plum-specific data.

Analytical approach. The review employs mixed-methods synthesis: (a) quantitative descriptive statistics (FAO production/trade time series); (b) qualitative thematic analysis of technological bottlenecks; (c) international benchmarking (Section 4.2); and (d) a Technology Deployment Index (Section 5, **Table 1**).

**Table 1:** Five-dimension international comparison matrix.

Dimension	Romania	Chile	Serbia	China	Europe/USA benchmark
Breeding: cultivars released in 20 years	~170	~30 (40 yr)	14	64 (70 yr)	8–10/yr
Breeding: annual release rate	8–10/yr	<1/yr	0.7/yr	~0.9/yr	8–10/yr
Value chain multiplier (farm-to-retail)	4–6×	6–8×	~2–3×	~2–3×	4–8×
Cold-chain penetration (pre-cooling)	~60%	>95%	<25%	<25%	~80%
Export phytosanitary certification rate	~80%	~95%	<10%	<10%	~70%
Processing scale (industrial)	Moderate	High	Low	Artisanal	Moderate
Brand international recognition	High	Moderate–High	Low	Low	High

## 2 Production and trade status of plum products

### 2.1 Global cultivation scale, yield and development trends

From 2021 to 2023, the global plum cultivation area remained relatively stable at 2,500–2,600 thousand hectares (kha): 2,589.4 kha in 2021, 2,629.1 kha in 2022, and 2,621.9 kha in 2023 (FAO, 2023) (**Table 2**). China dominates global production, with a 2023 cultivation area of 2,009 kha—accounting for over 75% of the global total. Serbia and Romania each have cultivation areas exceeding 50,000 hectares, while Russia, Bosnia and Herzegovina, and Turkey each surpass 20,000 hectares, reflecting their regional competitive advantages (FAO, 2023).

**Table 2:** Global plum cultivation area and yield from 2021 to 2023 (Unit: 1,000 hectares, 10,000 tons).

Year	Global cultivation area	China’s cultivation area	Global yield	China’s yield	China’s share of global yield
2021	2589.4	1994.3	1232.35	692.06	56.16%
2022	2629.1	2006.2	1256.43	693.23	55.17%
2023	2621.9	2010.8	1248.98	690.14	55.25%

Data definitions: FAO defines cultivation area as the area under permanent or temporary fruit cultivation regardless of bearing status; harvested area is the area from which at least one crop was harvested. This review favors harvested area for yield calculations. Global figures encompass FAOSTAT code 0235 (‘Plums and sloes’) including *P. salicina, P. domestica, P. cerasifera*, and related species. When referring specifically to *P. salicina*, this is explicitly stated. Units follow FAO convention: thousand hectares (kha) for area, million tons (Mt) for production.

This review focuses on the 2021–2023 period for three principal reasons: (i) it captures the full operational period following China’s formal 2020 Rural Revitalization strategic framework under the Central No. 1 Documents; (ii) it encompasses the global COVID-19 pandemic’s disruption of agricultural supply chains and the subsequent normalization phase, providing a coherent snapshot of post-pandemic industry adjustment; and (iii) FAOSTAT reports complete and validated production statistics for all member nations through 2023, making this the latest period of globally consistent data availability (FAO, 2023). Longer-term historical trends (5-year or 10-year periods) are referenced where informative for contextualizing structural shifts.

Global annual plum output stabilized at 12.3–12.6 million tons (Mt): 12.3235 Mt in 2021 (average yield: 4.76 t/ha), 12.5643 Mt in 2022 (4.77 t/ha), and 12.4898 Mt in 2023 (4.76 t/ha) (FAO, 2023). China maintained its leading position with 6.8888 Mt in 2023 (over 55% of global output), followed by Romania (645,100 tons); Chile, Serbia, and Turkey each produce less than 500,000 tons.

Varietal characteristics and utilization patterns vary distinctly across countries (FAO, 2023). For Serbia: It is renowned for indigenous varieties including ‘Nada’, ‘Crvena Ranka’, and the ‘Čačanska’ series. Unique varieties such as ‘trnovača’ and ‘požegača’ are used to produce national brandy (rakija), while sun-dried prunes from ‘Čačanska’ varieties are internationally recognized as the “European Preserve Kings”. For Romania: It combines traditional varieties (‘Tuleu gras’, ‘Grase romanesti’) with new cultivars (‘Carpatin’, ‘Centenar’) to optimize its industrial structure. For Chile: It leverages the Southern Hemisphere’s counter-seasonal advantage, with ‘Angeleno’, ‘Candy red’, and ‘Sweet Mary’ dominating exports; ‘D’Agen’ meets processing needs, while ‘Sweet Pekeetah’ targets high-end markets. For Turkey: Diverse varieties (*e.g*., ‘Papaz Erik’ for salt pairing, Japanese plums for fresh consumption, and blackberry plums for jam production) support global supply chains (**Table 3**).

**Table 3:** Characteristics of major plum varieties and their uses in main countries.

Country	Main varieties	Usage characteristics
Serbia	‘Nada’, ‘Crvena Ranka’, ‘Čačanska’ series, etc.	Local varieties are used in brandy (‘rakija’) production. Sun-dried plums of the ‘Čačanska’ variety are known as the “King of European Preserves”.
Romania	‘Tuleu gras’, ‘Grase romanesti’, ‘Carpatin’, ‘Centenar’, etc.	Combining traditional and new varieties to optimize the industrial structure.
Chile	‘Angeleno’, ‘Candy red’, ‘Sweet Mary’, ‘D’Agen’, ‘Sweet Pekeetah’, etc.	Leveraging the counter-seasonal advantage of the Southern Hemisphere for exports. ‘D’Agen’ is used for processing, and ‘Sweet Pekeetah’ targets high-end markets.
Turkey	‘Papaz Erik’, Japanese plums, blackberry plums, etc.	‘Papaz Erik’ (green-skinned, paired with salt), Japanese plums (for fresh consumption), blackberry plums (for jam production).

This production structure reveals a striking geographical concentration: the top five producing countries (China, Serbia, Romania, Chile, and Turkey) collectively account for approximately 75% of global plum and sloe output, with China alone contributing 55.3% (6.89 Mt of 12.49 Mt global total in 2023). However, production concentration does not equate to value concentration: Serbia, with only 5.4% of global output volume, achieves significantly higher per-hectare economic returns through integrated prune processing and branded export products, while Chile, with approximately 3.2% of global output, commands premium prices in Northern Hemisphere off-season markets through certified quality assurance and cold-chain logistics. This divergence between volume dominance and value marginalization constitutes the fundamental structural challenge underpinning China’s trade asymmetry, analyzed in detail in Section 2.3.3 below.

The global plum industry presents a development pattern characterized by “China-led large-scale production, with specialized contributions from other nations” (FAO, 2023). China retains quantitative dominance in both cultivation area and output, while Serbia, Romania, Chile, and Turkey have established differentiated competitiveness through specialty varieties, cultural branding, and advanced processing. Technological innovation, brand development, in-depth processing, and international market penetration are core drivers of industrial value enhancement, driving the industry toward globalization, diversification, and premiumization.

### 2.2 Cultivation scale, yield and output value in China

China, the world’s largest plum producer, has a cultivation distribution across Northwest, North, Northeast, East, and Southwest China, with each region developing regionally adapted unique varieties (FAO, 2023). Its harvested area grew steadily from 2021 to 2023, reaching 1,994.3 thousand hectares (kha) in 2021, 2,006.2 kha in 2022, and 2,010.8 kha in 2023. Annual output remained stable at approximately 6.9 million tons (Mt), recording 6.9206 Mt (2021), 6.9323 Mt (2022), and 6.9014 Mt (2023).

### 2.3 Import-export dynamics in China

#### 2.3.1 Trade scale and trends

In 2022, China’s total plum imports and exports amounted to 167,200 tons, valued at $379 million—of which 126,500 tons were imported ($323 million) and 40,700 tons exported ($56 million). Compared with the 2015 historical baseline, total volume and value increased by 167.83% and 128.2%, respectively, with respective compound annual growth rates (CAGRs) of 15.11% and 12.51%. Data from January–August 2023 showed continued expansion, with exports rising to 54,700 tons.

From 2018 to 2022 (a five-year period), imports grew from 54,900 tons to 126,500 tons (+130.4%), while exports increased from 34,100 tons to 40,700 tons (+19.46%). Net imports surged from 20,800 tons (2018) to 85,800 tons (2022), and the trade deficit expanded from $86 million to $267 million.

Import unit price rose from $2.35/kg (2018) to $2.93/kg (January–August 2023, +24.7%), whereas export unit price fell from $1.25/kg to $1.01/kg (-19.6%). In 2022, fresh plum import unit prices were twice that of exports, while prune import prices were 22.57% higher than export prices (FAO, 2023) (**Table 4**).

**Table 4:** Import and export of plums in China from 2018 to 2022 (Unit: tons, ten thousand USD).

Year	Import volume	Export volume	Net Import volume	Trade deficit	Import unit price (USD/kg)	Export unit price (USD/kg)
2018	54900	34100	20800	8600	2.35	1.25
2022	126500	40700	85800	26700	2.93	1.01

#### 2.3.2 Trade structure

In 2022, China’s plum product trade exhibited structural import dependence, with distinct patterns across product categories (FAO, 2023). For fresh plums (including sloes), 103,500 tons were imported (valued at $270 million), while exports reached 38,700 tons ($52 million), corresponding to a net import of 64,800 tons and a trade deficit of $218 million—reflecting domestic supply shortages. For prunes (dried plums), imports stood at 23,000 tons ($53 million) versus 2,000 tons exported ($4 million), with a net import of 21,000 tons and a $49 million deficit, indicating reliance on foreign supplies (**Table 5**).

**Table 5:** Trade structure of Chinese plum products in 2022 (Unit: tons, ten thousand USD).

Product type	Import volume	Import value	Export volume	Export value	Net import volume	Trade deficit	Proportion of import volume	Proportion of export volume
Fresh plums and sloes	103500	27000	38700	5200	64800	21800	82%	95%
Prunes and dried plums	23000	5300	2000	400	21000	4900	18%	5%

Fresh plum products dominated both trade flows, representing 82% (volume) and 84% (value) of total imports, and 95% (volume) and 93% (value) of total exports. Geographically, imports were concentrated in coastal regions: Guangdong province, Shanghai, and Zhejiang province accounted for 52.64%, 17.08%, and 10.9% of total imports, respectively, with a combined share of 80.62%. For exports, Yunnan province led with 51.8%, followed by Guangdong province (12.41%) and Guangxi Zhuang Autonomous region (11.81%), collectively contributing 76.02% of total exports; Vietnam was the primary destination, accounting for 72.06% of export volume and 67.24% of export value. Notably, Chile supplied 84.45% of China’s fresh plum imports, highlighting heavy reliance on Southern Hemisphere producers.

This export concentration is attributable to three interlocking factors. First, geographic proximity reduces logistics costs and cold-chain transit times, which is critical for a climacteric fruit with a limited postharvest window. Second, the RCEP (Regional Comprehensive Economic Partnership), which entered into force in January 2022 among China, Vietnam, and key ASEAN members, progressively eliminates tariffs on more than 90% of goods varying timeframes, substantially lowering bilateral trade costs for agricultural products (ASEAN Secretariat, 2022). RCEP’s Rules of Origin provisions have demonstrably facilitated agri-export growth, with Vietnam’s fruit exports to China expanding significantly under RCEP tariff concessions (Xinhua Net, 2024a). Third, Southeast Asian markets accept quality grades that diverge from EU/US phytosanitary standards, permitting the commercialization of fruits that do not meet stringent EU pesticide residue limits (EU Regulation 396/2005), thereby creating a de facto market segmentation that reinforces the export–import asymmetry.

Despite contributing 54% of global plum production (FAO, 2022) and 23% of global plum imports (2023), China’s export influence remains constrained—concentrated primarily on Southeast Asia—revealing a “high import, low export” imbalance.

#### 2.3.3 Structural determinants of the import–export asymmetry

China’s paradoxical position as both the world’s largest plum producer (6.89 Mt, 55.3% of global output) and a net importer with a USD 267 million trade deficit (2022) reflects five interlocking structural deficiencies, each empirically documented in the trade economics literature. (i) Cultivar quality and varietal composition: A disproportionately large share of Chinese output derives from small-fruited (30–40 g/fruit), short-shelf-life cultivars poorly adapted to international fresh-fruit chains. Chinese domestic varieties average 5–7 days’ commercial shelf life under ambient conditions, compared with ‘Angeleno’ (65–75 g, 20-day shelf life) from Chile, the dominant supplier to China’s import market. Fewer than 10 of China’s 64 registered varieties meet the ≥60 g and ≥14-day firmness thresholds required for EU/US supermarket distribution standards, creating a structural quality ceiling that domestic breeding programs have failed to breach despite 70 years of formal variety release activities. (ii) Consumer preferences and market structure: Domestically, Chinese consumers—particularly in tier-2 and tier-3 cities constituting the majority of plum purchasers—prioritize sweetness, acidity balance, and cultural/traditional variety identity over uniform appearance and size. This demand heterogeneity has encouraged breeders and extension services to optimize eating quality traits at the expense of postharvest durability and export compatibility, creating a path-dependent lock-in that constrains export-oriented varietal development. (iii) Postharvest infrastructure deficit: China lacks the centralized, prescriptive cold-chain logistics infrastructure that enables Chilean and South African exporters to maintain >95% fruit quality integrity at point-of-sale in export markets. Pre-cooling is applied to less than 25% of domestically produced plums before transport, and cold-chain penetration during distribution to export ports remains below 40% for plum-specific cargo. In contrast, Chile’s export sector requires hydro-cooling within 4 hours of harvest and continuous temperature monitoring at 0–2°C throughout the supply chain—protocols that are technically feasible but economically prohibitive for fragmented Chinese smallholder systems without consolidation or cooperative aggregation. (iv) Market segmentation and value chain positioning: China’s plum exports are disproportionately represented in lower-value market segments (Southeast Asia, priced at USD 0.80–1.10/kg), while high-value EU/North American markets (priced at USD 3.00–5.00/kg for equivalent quality grades) are effectively inaccessible to most Chinese producers because current product quality does not conform to the MRL and quality standards required. This creates a de facto two-tier global market in which Chinese producers are locked into price competition in lower-value segments. (v) International competitiveness asymmetry: Chile, the dominant supplier to China’s import market (84.45% of fresh plum imports), achieves a farm-to-retail value multiplication factor of approximately 6–8× through rigorous GAP/GLOBALG.A.P. certification, HIF cold-chain logistics, and brand positioning. Chinese producers can only compete by achieving comparable quality standards or by occupying seasonal windows that Southern Hemisphere producers cannot fill, necessitating strategic investment in protected cultivation and off-season production technologies.

The concentration of exports in Southeast Asia rather than higher-value EU or US markets reflects the operation of binding non-tariff barriers, principally Sanitary and Phytosanitary (SPS) measures and Technical Barriers to Trade (TBT). The EU enforces maximum residue limits (MRLs) for more than 1,000 pesticide active substances under Regulation (EC) No 396/2005, with default MRLs of 0.01 mg/kg for unregistered substances. Chinese plum exports face documented rejections at EU border inspection posts due to pesticide residues and incomplete phytosanitary certification (Dong and Jensen, 2007). Empirical estimates indicate that SPS measures will suppress China’s agricultural exports in the short term (Hua et al., 2024). Similarly, the USDA Animal and Plant Health Inspection Service (APHIS) maintains a strict import compliance regime. Overcoming these barriers requires systematic pre-export residue monitoring, adoption of Good Agricultural Practices (GAP) at the orchard level, and internationally recognized certification systems.

## 3 Necessity of accelerating plum industry development in the new era

### 3.1 Driven by national strategic demands

#### 3.1.1 Supporting rural revitalization

As a pillar industry in mountainous and hilly regions, China’s plum sector contributes significantly to growers’ income and regional economic development. Cultivated across more than 20 provinces, it yielded 6.8888 million tons in 2023 (FAO, 2023). Geographical indication (GI) products such as ‘Sanhua plum’ and ‘Furong plum’ have not only boosted employment but also driven regional growth.

Nevertheless, challenges including varietal homogeneity, low premium fruit rates, and truncated value chains hinder industrial efficiency enhancement. Technological innovation is critical to addressing these issues, covering three key areas: varietal improvement for developing high-value cultivars, digital agriculture adoption for precision management, and advances in deep processing to extend the industrial chain value.

The 2023 Central No. 1 Document’s focus on new agricultural industries supports this industrial transformation. A typical example is Chunfeng village in Yibin: via the “Party branch + enterprise + farmer” model, the village leveraged the branding of ‘Mao’erwan Chunfeng plum’ and integrated tea and flower industries, achieving an annual output value of 3.5 million yuan. Additionally, “14th Five-Year Plan” policies promoting agricultural parks, industrial integration, and cooperative development have accelerated the plum industry’s modernization. Backed by technological upgrading and policy support, the sector is positioned as a key driver of rural revitalization.

#### 3.1.2 Advancing seed industry revitalization

China’s plum seed industry faces several challenges, including limited varietal innovation, inefficient germplasm resource utilization, and underdeveloped industrial value chains (Liu et al., 2019). Revitalizing this sector requires integrated progress across three core dimensions. First, for germplasm conservation and utilization: China holds nearly 1,000 *Prunus* germplasm accessions (Liu et al., 2019), yet their utilization is relatively limited—biotechnological tools like molecular marker-assisted breeding (MAS) offer great potential to accelerate the identification of genes linked to stress resistance and environmental adaptability. Second, in breeding innovation: Modern biotechnologies (*e.g*., gene editing, genomic selection) have facilitated the development of cultivars with enhanced traits (disease resistance, cold tolerance, extended shelf life), as seen in varieties such as ‘Guomei’ and ‘Guoli’ (Xu et al., 2015; Zhang et al., 2019). Third, in advancing cultivation and processing: Adopting precision agriculture, digital monitoring systems, and advanced deep processing technologies (*e.g.*, vacuum sugaring, juice brewing) boosts productivity and enriches product diversity.

This multi-dimensional strategy enhances varietal diversity, strengthens market competitiveness, and improves industrial efficiency—ultimately consolidating China’s standing in the global plum industry (Gong et al., 2024; Han et al., 2025; Uztürk and Büyüközkan, 2024).

#### 3.1.3 Addressing climate change and resource constraints

Plum cultivation is highly vulnerable to climate change: rising temperatures drive northward shifts in growing zones, precipitation variability disrupts growth cycles, and extreme weather events (*e.g*., late spring frosts, heavy rains) cause substantial losses (Guerrero et al., 2024). Nevertheless, the industry holds significant adaptive potential to mitigate these adverse impacts via adopting stress-resistant varieties, implementing diversified cultivation practices, and advancing technological upgrades (Khundrakpam and Kotiyal, 2024; Jyoti et al., 2025).

Key technological solutions include:

Stress-resistant breeding: Focused on developing cold-, drought-, and disease-resistant cultivars using modern breeding techniques;
Precision agriculture: Incorporating IoT-based monitoring systems to optimize water and nutrient management, plus drone-based pest control;
Protected cultivation: Deploying greenhouses and rain-shelter systems to reduce weather-related risks and enable off-season production;
Mechanization: Developing and applying small-scale machinery adapted to mountainous orchards to alleviate labor shortages (Mei et al., 2025).

These innovations collectively boost the plum industry’s climate resilience, advance its sustainable development, and enhance its industrial competitiveness.

#### 3.1.4 Breaking technological bottlenecks

The economic viability of these solutions for smallholder farmers requires explicit cost–benefit analysis. Multi-span greenhouses entail construction costs of ¥350,000–500,000 per mu, offset by off-season price premiums of 40–80% and yield increases of 25–35% (China Agricultural University Agricultural Planning Service Platform, 2025). Smart orchard monitoring costs ¥15,000–30,000 per mu for installation, reducing water and fertilizer inputs by 15–20% and saving approximately ¥1,200 per farmer per growing season in labor (Chongqing municipal people’s government, 2025). Biological pest control achieves cost equivalence with chemicals within 3–5 years when factoring in MRL compliance benefits and avoided resistance management costs.

Key breakthrough areas for China’s plum industry include fruit quality enhancement, breeding strategy reform, and international market adaptation: fruit quality enhancement focuses on precision breeding, via gene editing and genomics targeting sugar-acidity balance, aroma, and texture to meet high-end market demands; breeding strategy reform is characterized by a shift from traditional lengthy hybridization to molecular marker-assisted breeding (MAS), genomic selection (GS), and gene editing for targeted trait improvement; and international market adaptation centers on developing varieties compliant with global standards for appearance, flavor, and shelf life, supported by green pest management and advanced cold chain logistics. Additionally, integrated smart orchard management and diversified deep processing further resolve industrial inefficiencies, enabling a quality-driven, globally competitive development model (Fang et al., 2020; Varshney et al., 2005; Liu et al., 2019).

### 3.2 Meeting upgraded market demands

#### 3.2.1 Consumer demand for high-quality products

Market trends in the plum industry reflect three evolving consumer demands—premium appearance, standardized flavor, and functional nutrition—driven by rising health consciousness, consumption stratification, and global supply chain advancements (Török et al., 2023; Myracle et al., 2018). To align with these trends, the industry requires technology-led innovation: varietal breakthroughs to tackle genetic homogeneity and over-reliance on foreign germplasm; adoption of precision cultivation, which replaces experience-based farming with data-driven management; and extension of the value chain beyond fresh fruit to high-value processed products. Currently, the industry faces bottlenecks including genetic homogeneity, inconsistent fruit quality from extensive farming, and insufficient processing depth (Jalili et al., 2023; Wang et al., 2021). Addressing these challenges demands targeted innovation in breeding, smart agricultural technologies, and integrated processing techniques to realize sustainable development.

#### 3.2.2 International market opportunities

Despite China’s plum industry holding a leading position in global production, its exports account for only 1–2% of total output—a limitation rooted in inconsistent product quality, a shortage of premium varieties, and deficiencies in cold chain and preservation technologies (FAO, 2022). International markets have a clear preference for large-fruited (≥60 g), vividly colored, and transport-resistant varieties, *e.g*., America’s hybrid ‘Kong long dan’ (fruit weight >80 g, vibrant skin) and Chile’s ‘Royal Gem’—traits often absent in Chinese plum varieties (Crisosto and Kader, 2000).

To address these challenges, technological solutions should prioritize breeding internationally compliant varieties via genomics and gene editing, and advancing cold chain logistics—including smart temperature-controlled systems and modified atmosphere packaging (MAP). Additionally, adopting commercial strategies (cross-border e-commerce, geographical indication branding) can further expand global market access, backed by strict adherence to international quality standards (Han et al., 2026, Savadi et al., 2021, Li et al., 2024; Liao et al., 2025).

## 4 Regional distribution and technology systems

### 4.1 Major production regions

**Figure 1:**
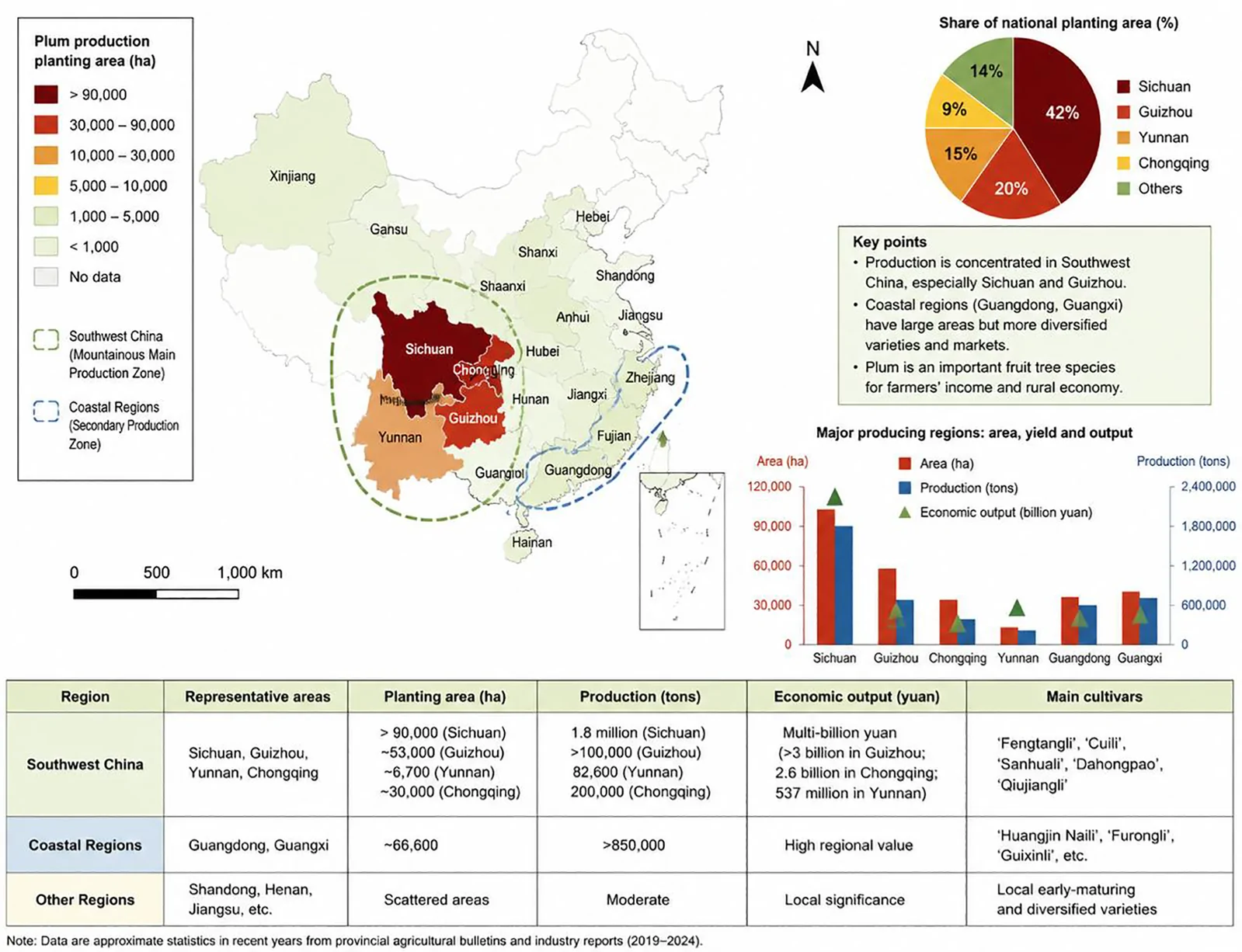
Geographic distribution and scale of China’s plum industry. This image presents the spatial distribution and production scale of the plum industry across major regions in China. It highlights the concentration of production in Southwest China (*e.g*., Sichuan, Guizhou, Yunnan, Chongqing), where mountainous agro-climatic conditions support large-scale cultivation. Key production indicators include planting area (hectares), yield (tons), and economic output (yuan). The image further contrasts these regions with coastal production zones (Guangdong, Guangxi), illustrating regional specialization and varietal diversity. The visualization emphasizes the uneven geographic distribution and the dominance of several flagship cultivars such as ‘Fengtangli’ and ‘Cuili’.

China’s plum cultivation spans diverse ecological zones, with core regions distributed across Northeast, Northwest, Southwest, and South/East Coastal China, each forming regionally adapted industrial layouts (**Figure 1**; **Table 6**).

**Table 6:** Regional distribution and production scale of China’s plum industry.

Region	Representative areas	Planting area (ha)	Production (tons)	Economic output
Southwest China	Sichuan, Guizhou, Yunnan, Chongqing	Large-scale (>90,000 ha in Sichuan)	Up to 1.8 million tons	Multi-billion yuan
Chongqing	Wushan, Wuxi	30,000	200,000	~2.6 billion yuan
Guizhou	Zhenning, Yanhe	~53,000	~100,000+	>3 billion yuan
Yunnan	Suijiang	6,667	82,600	537 million yuan
Coastal Regions	Guangdong, Guangxi	~66,600	>850,000	High regional value

Northeast China, defined by a cold climate, has developed distinct cultivation patterns. In Liaoning province, Yingkou’s ‘Dahongpao’ plum covers 1,000 hectares, with an annual output of 3,500 tons and output value of 50 million yuan; Jiuzhai town cultivates 166.67 hectares of ‘Heiba’ and ‘Xingyun’ varieties, yielding 7,000 tons. Jilin province had 18,133 hectares of plum orchards in 2024, producing 472,500 tons and a total output value of 1.28 billion yuan—key varieties include Shulan’s ‘Ganwan’ plum (93 hectares, 4,000 tons) and Jiutai’s late-ripening varieties (over 400 hectares, 30,000 tons). Heilongjiang province focuses on cold-resistant varieties such as ‘Suili 3’ (2,333 hectares, 33 t/ha) and ‘Mufeng’ (20 hectares), well-suited to frigid conditions.

Northwest China, featuring high solar radiation and low precipitation, centers its plum industry on Xinjiang, which specializes in European plums (*Prunus domestica*). Jiashi County accounts for 40% of China’s prune cultivation area (36,666 hectares and contributes 60% of national output; Huocheng’s wild cherry plum covers 4,000 hectares, yielding over 4,000 tons annually. Varieties like ‘Weidi’ (113 hectares, 15 t/ha) and ‘Kong long dan’ thrive in region-specific conditions (China Daily, 2023-09-22; People’s Daily Online, 2023-09-19).

Southwest China, with a mountainous climate, supports a robust plum industry across multiple provinces. In Chongqing, Wushan ‘Cuili’ plum covers 20,000 hectares (140,000 tons, 2.1 billion yuan), while Wuxi’s late-ripening plum occupies 10,000 hectares (60,000 tons, 500 million yuan). Sichuan province has 92,733 hectares of plum orchards, producing 1.8062 million tons (second only to citrus)—key varieties include Maoxian ‘Qiangcuili’ (6,666 hectares, 200,000–250,000 tons) and Pingshan ‘Yinhongli’ (11,333 hectares, 160,000 tons) (Xinhua, 2025-07-10; Media OutReach, 2024-03-14; iChongqing, 2024-09-04). Guizhou province’s industry is dominated by ‘Fengtangli’ (53,333 hectares, 65% of provincial total), ‘Kongxinli’ (12,000 hectares, 15%), and other varieties (20%); Zhenning ‘Fengtangli’ covers 14,673 hectares (59,700 tons, 3.001 billion yuan, 60% of national output), and Yanhe ‘Kongxinli’ spans 6187 hectares across 11 towns (45,000 tons, 70 million yuan). Yunnan province’s Suijiang ‘Banbianhong’ plum—with a 70-year cultivation history—covers 6,667 hectares (82,600 tons, 537 million yuan) (Guizhou Government/China Daily, 2024-07-17).

The South and East coastal regions have developed characteristic plum industries based on local conditions. In Guangxi, Babu ‘Xindu Sanhua plum’ covers 6,600 hectares (50,000 tons in 2023, projected 135,000 tons by 2025). Guangdong province has 60,000 hectares of plum orchards, producing 806,300 tons in 2022 (ranking 5th among local fruits)—led by Xinyi ‘Sanhua plum’ (23,333 hectares, 359,700 tons, 5.047 billion yuan) and Wengyuan’s local varieties (2,400 hectares, 35,000 tons, 70 million yuan). Fujian province had 27,100 hectares of plum orchards in 2023, producing 414,100 tons (ranking 1st among deciduous fruits); Yongtai County is a major ‘Furong plum’ producer (8,667 hectares), and Tantou Town yields 24,600 tons from 1,987 hectares. Zhejiang province features regional specialties like Shengzhou ‘Taoxingli’ (1,667 hectares, 30,000 tons, 300 million yuan) and Tongxiang ‘Zuili’ (133 hectares, 300,000 yuan/he) (GDToday Editor, 2025-04-24; FreshPlaza.com, 2025-04-25; GDToday Editor, 2025-06-11).

### 4.2 International benchmarking: China in comparative perspective

#### 4.2.1 Quantitative benchmarking matrix

Gap interpretation: China’s most severe deficits are breeding velocity, cold-chain logistics, and export certification. These three domains must be addressed concurrently if China is to shift from volume-dominated exports toward high-value, internationally competitive trade. **Table 1** consolidates the five-dimension international comparison into a single quantitative matrix.

**Figure 2:**
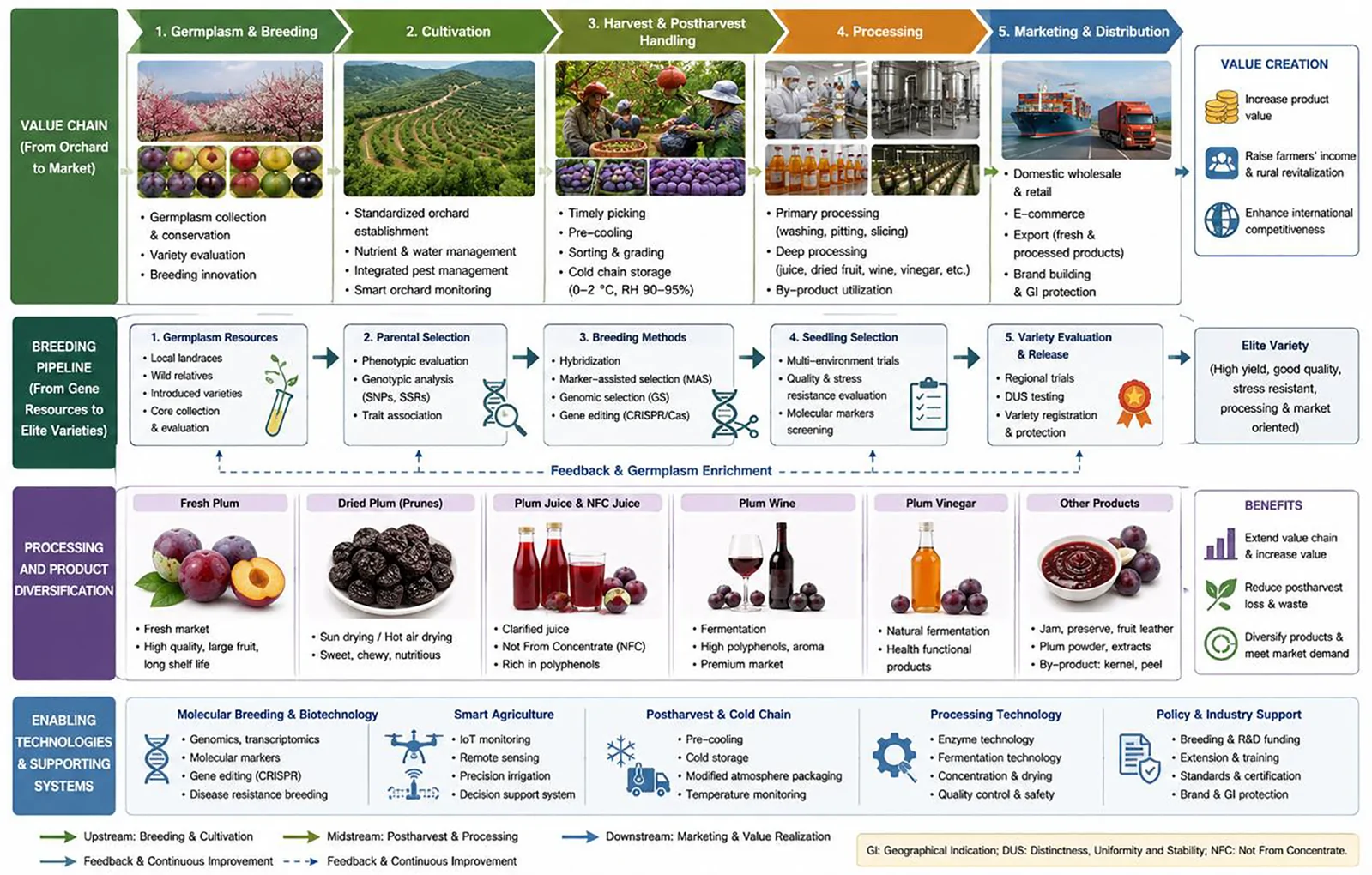
Value chain and breeding-processing pipeline of China’s plum industry. The complete industrial operation logic from germplasm innovation to terminal market value realization is systematically summarized in **Figure 3**, which decomposes the whole plum value chain, breeding technical pipeline, diversified deep-processing products and multi-dimensional supporting technology systems, providing a holistic framework for subsequent analysis of regional industry differences, international gaps and technical bottlenecks.

A global comparison reveals the magnitude of China’s structural gaps compared with Chile, Serbia, Romania, and Turkey across five analytical dimensions. (1) Breeding strategies and variety release rates: European programs released approximately 170 new plum cultivars over the past two decades through coordinated multi-institutional consortia—Romania contributing 38, Serbia 14, Germany 28, and Italy 13—derived from approximately 250,000 hybrid combinations, with 80% of breeding focused on *P. domestica* (Butac et al., 2012; Sottile et al., 2022). The United States, via the USDA-ARS program, has released nearly 30 improved cultivars in the past 40 years, including the virus-resistant transgenic ‘Honey Sweet’ (Scorza et al., 2016). In contrast, China’s 64 registered varieties, accumulated over seven decades, trace to a remarkably narrow parental base and average fewer than one new release per year, markedly lower than the European rate of 8–10 cultivars per year. (2) Value chain development and value multiplication: Chilean exporters achieve a farm-to-retail value multiplication factor of 6–8× through rigorous GAP/GLOBALG.A.P. certification, hydro-cooling within 4 hours of harvest, and 0–2°C continuous cold-chain logistics. China’s value chain achieves only 2–3×, constrained by price competition, lack of standardized grading, and fragmented distribution. (3) Export competitiveness and phytosanitary compliance: Chile commands >84% of the ad valorem market share in China’s import market despite Southern Hemisphere counter-seasonality, because of consistent quality and phytosanitary compliance—a structural asymmetry Chinese producers cannot overcome without comparable standards. (4) Processing technology and industrial scale: Serbia leads global prune production with industrial-scale sun-drying facilities (capacity >100,000 t/year) and branded export products. China’s processing remains predominantly artisanal, with limited industrial-scale dehydration or fermentation infrastructure. (5) Branding strategies and geographical indication: European plums benefit from PDO/PGI protection and cultural branding (*e.g*., ‘Pruneau d’Agen’ France, ‘Šljivovica’ Serbian plum brandy with EU Geographical Indication status). China’s GI system has limited international recognition, and brand-driven price premiums in export markets remain elusive (**Figure 2**; **Table 7**).

**Table 7:** International comparison of plum breeding systems.

Country/region	Cultivars released (20 yrs)	Annual release rate	Breeding strategy	Key Features
Europe	~170	8–10/year	Multi-institutional hybridization	High diversity, strong coordination
USA	~30 (40 yrs)	<1/year	Advanced biotech (*e.g*., transgenic)	Virus resistance
China	64 (70 yrs)	~0.9/year	Local × foreign hybridization	Narrow genetic base

## 5 Technological applications and bottlenecks in the plum industry chain

### 5.1 Key technical dimensions of China’s plum industry

#### 5.1.1 Breeding

China’s plum breeding program, initiated in the 1950s–1960s, has resulted in 64 registered varieties, with cold hardiness, high yield, and superior quality as core objectives (Liu et al., 2023, 2018). Northeast provinces contributed 57.81% of these varieties, followed by Xinjiang and Shanxi. Key milestones include: 1) 1956, when ‘Liuhao Li’ laid the foundation for the ‘Changli’ series; 2) the 1960s–1970s, when Heilongjiang and Jilin developed cold-hardy varieties (*e.g*., ‘Suilinghong’, ‘Changli 7’) via seed selection; 3) subsequent decades, when hybridization integrated with seed selection produced ‘Changli 15’ and ‘Longyuan Qiuli’ (cold-hardy, early-maturing, high-yielding); and 4) 2000s to present, when multi-institutional collaboration yielded varieties like ‘Qiuxiang Li’ and ‘Bashan Cuili’ primarily through local variety selection or intra-species crosses. Current breeding strategies focus on “East cross West”, which actually indicates the “local × foreign variety” hybridization, aiming to integrate local varieties adaptability and disease resistance with foreign varieties superior appearance and storage traits (Liu et al., 2018, 2023), exemplified by Liaoning’s ‘Guomei’ and ‘Guofeng’, which embody these combined characteristics.

The severity of this genetic bottleneck is apparent only in international comparison. Over the past two decades, European breeding programs alone have released approximately 170 new plum cultivars, Romania contributing 38, Serbia 14, Germany 28, and Italy 13, derived from approximately 250,000 hybrid combinations (Butac et al., 2012; Sottile et al., 2022). In the United States, the USDA-ARS program has released nearly 30 improved cultivars in the past 40 years, including the PPV-resistant transgenic ‘Honey Sweet’ (Scorza et al., 2016). Chile, while dominated by hybrid cultivars of foreign origin, actively maintains a variety improvement pipeline linked to Southern Hemisphere counter-seasonal market access. In contrast, China’s 64 registered varieties, accumulated over seven decades of breeding, trace to a remarkably narrow range of parental lines. The rate of new cultivar release averages fewer than one per year, markedly lower than the European rate of 8–10 cultivars per year across formal programs.

Identified bottlenecks: Despite seven decades of breeding effort, China’s 64 registered varieties trace to a remarkably narrow range of parental lines (predominantly ‘Formosa’ and ‘Liuhao Li’), leading to insufficient breakthroughs in key traits such as storage performance, processing suitability, and stress tolerance. Additionally, there is a severe lag in specialized variety development—particularly lacking cultivars for deep processing (*e.g*., stable juice color or wine-specific flavor profiles) and varieties adapted to extreme environments (saline-alkali land, frigid regions). These constraints limit the industry’s ability to meet evolving market demands for premium, processed, and climate-resilient plum products.

**Figure 3:**
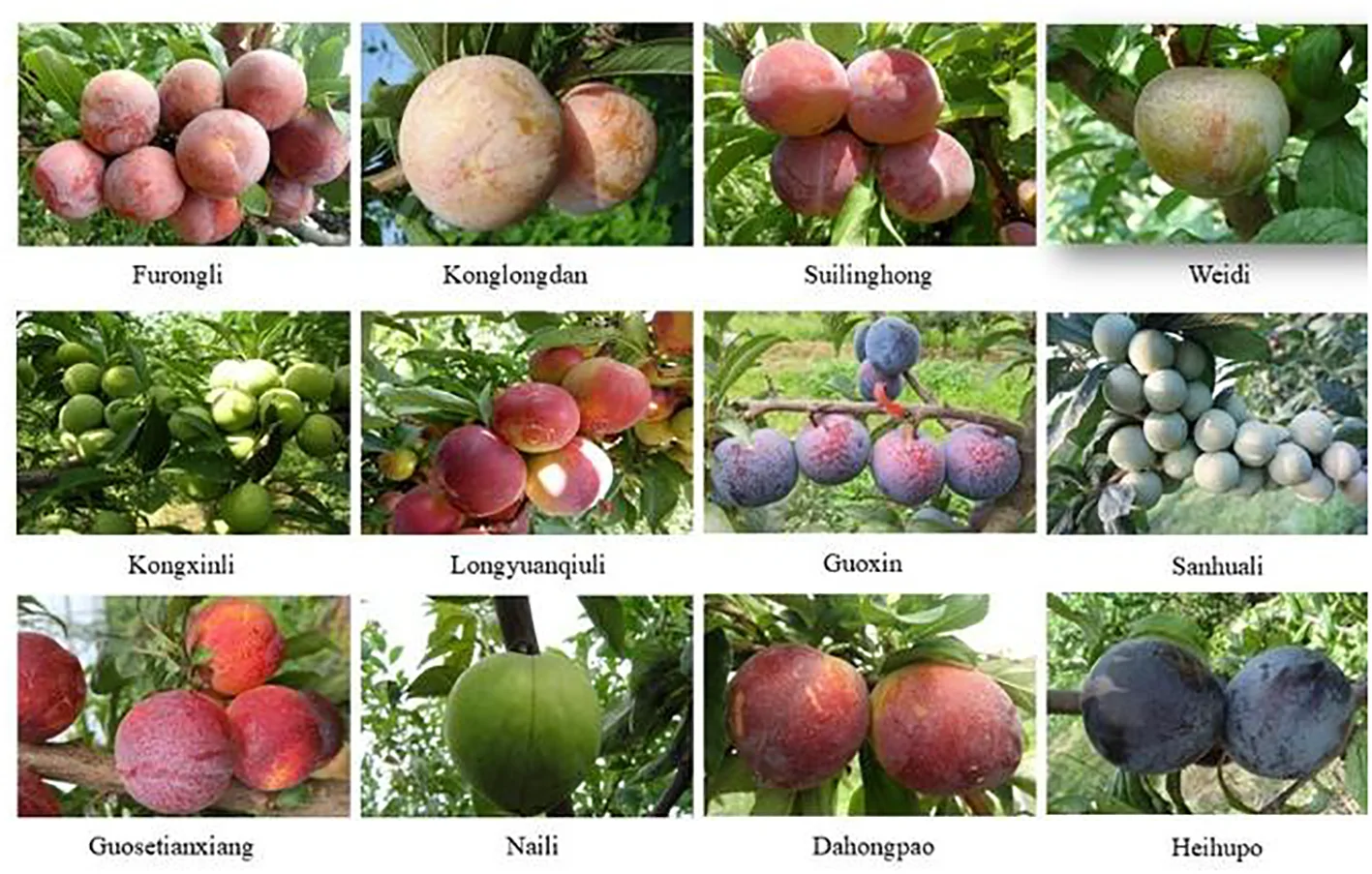
Morphological diversity of Chinese plum cultivars. This image displays representative fruit morphology of major Chinese plum cultivars. Each panel corresponds to a specific cultivar (*e.g*., Furongli, Kongxinli, Sanhuali), showing on-tree fruit characteristics including skin color (green to black spectrum), fruit shape, size, and cluster morphology. The image serves as a visual germplasm reference, illustrating the phenotypic diversity of Chinese plum varieties and supporting comparative analysis in breeding and classification studies.

Quantitatively, the breeding performance gap is stark: China’s annual rate of new cultivar release averages 0.9 varieties per year over 70 years, compared with Romania’s 1.9 per year (38 cultivars/20 years), Serbia’s 0.7 per year (14 cultivars/20 years), and the pan-European consortium average of 8–10 per year across formal programs (Butac et al., 2012). Moreover, the genetic contribution of each new Chinese variety is constrained by the narrow parental base: 57.81% of all registered varieties trace to Northeast progenitors, with ‘Formosa’ and ‘Liu hao Li’ appearing in the pedigrees of over 60% of commercial cultivars (Liu et al., 2023). This genetic monopolization limits the heterosis potential achievable through intra-specific crossing and underscores the strategic necessity of the ‘East Cross West’ hybridization strategy (**Figure 3**).

#### 5.1.2 Cultivation

Research on plum tree physiological mechanisms has laid the foundation for cultivation practices, yet challenges remain in adaptive regulation, quality enhancement, and stress management (Ma and Liu, 2020). In photosynthetic physiology, varietal differences exist in midday depression: ‘Heihupo’ and ‘Suili 3’ exhibit this phenomenon, while ‘Dashi Zaosheng’ does not under protected cultivation (Zhang, 2005; Yang et al., 1998; Yu et al., 2004). For fruit quality formation, late-maturing varieties show better sugar accumulation, with malic acid peaking in young fruits (Liu et al., 2016, Zhao et al., 2004). Regulation of PEPC and NADP-ME enzymes informs precision fertilization (organic manure increases sugar content and reduces acidity) and growth regulator application (CPPU and GA₃ improve soluble solids) (Ou et al., 2006; Wang et al., 2018b). Ethylene combined with GA₃ reduces fruit cracking, but universal protocols for different varieties are lacking (Cui et al., 2006). In stress physiology, cold hardiness correlates with branch electrolyte leakage, soluble sugar content, and protective enzyme activity; exogenous salicylic acid and ABA enhance flower cold tolerance (Li et al., 2017). In drought-prone regions, root moisture conservation techniques are used, and ‘Ziye Li’ salt tolerance mechanisms inform plum cultivation in urban and saline-alkali lands (Hu et al., 2010). Overall, plum cultivation exhibits “systematic basic research yet uneven technical application,” requiring improved regional adaptation and standardized “superior variety + superior method” systems (Ma and Liu, 2020).

Identified bottlenecks: The industry lacks precision regulation systems, with inconsistent application of environment-specific technologies (*e.g*., protected cultivation parameters, drought and salt damage repair measures). A critical disconnect exists between molecular breeding and cultivation practices, genetic markers are rarely integrated with agronomic measures (*e.g*., fertilization, hormone regulation) to improve quality and stress resistance. Standardization remains incomplete, with poor coordination between varieties and regional cultivation practices, plus vulnerability to extreme weather from over-reliance on traditional experience rather than data-driven protocols.

#### 5.1.3 Plant protection

**Figure 4:**
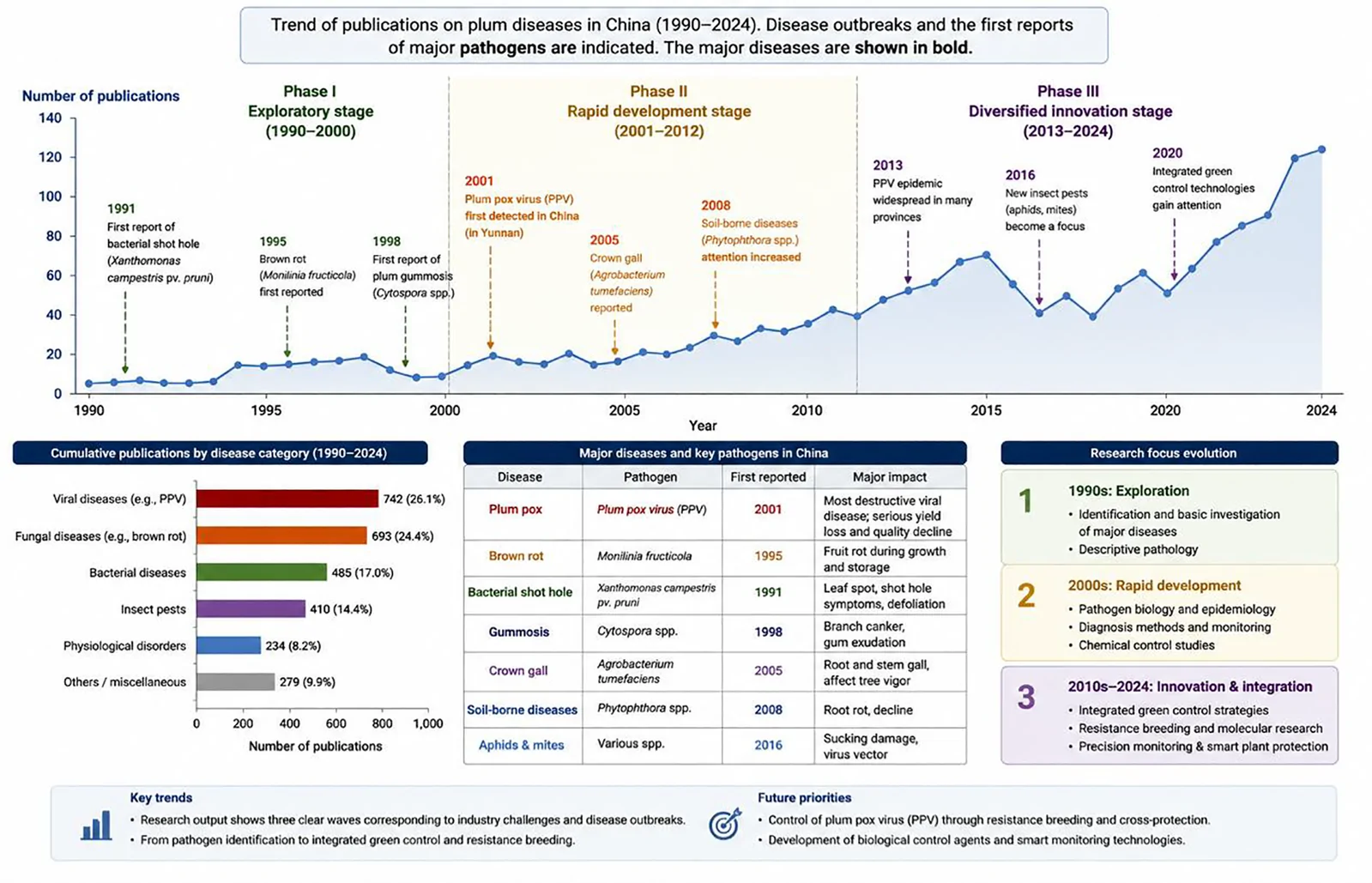
Research trends in plum diseases in China. This image illustrates temporal trends in plum disease research in China over the past three decades. It presents the number of publications per year, identifies major disease outbreaks (*e.g*., plum pox virus), and marks the first reported occurrences of key pathogens. The image reveals cyclical research intensity and highlights emerging priorities in disease control and resistance breeding.

Plum production faces growing pest and disease pressures amplified by climate change, with key threats including bacterial shot hole, brown rot, gummosis, crown gall, soil-borne diseases, and insect pests (*e.g*., Grapholita funebrana, aphids, spider mites) (Liu, 2008; Mu and Meng, 2022; Zhao and Zhou, 2023, Zhang et al., 2025b). Among these, the quarantine plum pox virus (PPV) poses the greatest threat (Cambra et al., 2006; Scorza et al., 2013). Critical limitations in plum plant protection include: 1) the absence of national research programs and specialized teams, leading to insufficient systematic understanding of plum-specific pests/diseases (Niu et al., 2019); 2) pest control mostly depending on experience-based practices or crop-borrowed technologies, lacking targeted solutions (Yang and Yao, 2020); 3) over-reliance on chemical pesticides (accounting for >70% of control methods), causing pesticide resistance and environmental issues (Wang et al., 2009; Zhu and Liang, 2024); 4) limited adoption of physical controls (*e.g*., light traps, color plates) by smallholders and low usage of biological agents (*e.g*., Bt, Bacillus subtilis; <10% of applications) (Niu et al., 2019); 5) smart plant protection in its infancy, with only pilot IoT-meteorological monitoring in Guangdong, immature AI diagnostic models, and no national pest-disease database (Bie et al., 2026); and 6) scarce resistant varieties, only ‘Guofeng 7’ (brown rot-resistant) and ‘Heibaoshi’ (bacterial shot hole-resistant) exist, while major local varieties (*e.g*., ‘Furong Li’, ‘Sanhua Li’) lack resistance traits (Zhong, 2018; Yang et al., 2023). Compared to international advancements (*e.g*., U.S. transgenic PPV-resistant varieties, Dutch automated Trichogramma production), China’s plum plant protection system needs comprehensive advancement to address these gaps (Goswami et al., 2017, Schäfer and Herz, 2020; Scorza et al., 2013, 2016) (**Figure 4**).

The ecological costs of this over-reliance on chemical pesticides are particularly acute in the mountainous regions where most plum orchards are concentrated. Continuous agrochemical application in these ecologically sensitive zones is associated with (i) soil microbial community disruption, reducing beneficial mycorrhizal populations and soil enzymatic activity (Wang et al., 2009; Zhu and Liang, 2024); (ii) pesticide residue accumulation in upper soil horizons, with potential leaching into headwater catchments supplying downstream agricultural and domestic water users; (iii) secondary pest outbreaks caused by the disruption of natural enemy populations following broad-spectrum insecticide applications; and (iv) impacts on above-ground biodiversity, with studies in China’s southwestern mountainous fruit orchards documenting declines in insect pollinator abundance correlated with spray frequency (Dicks et al., 2021). These impacts undermine ecosystem service provision critical for plum productivity (pollination, natural pest regulation) and create long-term soil quality degradation. The transition to integrated pest management is therefore not only an agronomic imperative but a prerequisite for sustaining the ecological base upon which long-term orchard productivity depends.

Identified bottlenecks: Beyond the over-reliance on chemical pesticides (>70% of control methods), the industry faces systemic deficiencies including: a shortage of resistant germplasm and immature genetic transformation systems; lack of plum-specific biological control agents (broad-spectrum products show unstable efficacy compared to international standards); costly mass-rearing of natural enemies (¥0.5 per individual) without automated production systems; dependence on imported elicitors (*e.g*., chitosan); and absence of commercialized pathogen detection kits, unlike the US market where Agdia has offered PPV kits for over 20 years. These gaps collectively constrain the transition to sustainable, export-competitive plant protection systems.

The Technology Deployment Index quantified in **Table 1** confirms this asymmetry: while chemical control achieves >70% adoption, biological control—despite demonstrated efficacy—remains below 10% deployment. Similarly, precision irrigation and IoT-based monitoring show availability rates above 60% but deployment rates under 25%, indicating that availability and adoption are decoupled by economic and institutional barriers rather than technological absence. In contrast, MAS-assisted breeding, while available at specialized institutions, has not been adopted by any commercial variety release program as of 2024.

The adoption barriers for biological control are structurally embedded in China’s smallholder production system. Mass-rearing of Trichogramma wasps (costing ¥0.5 per individual, or ¥300–500 per mu per season) requires centralized production facilities, cold-chain distribution of living insects, and farmer training in release timing—none of which are available through current extension channels. In contrast, chemical pesticides are distributed through an established retail network, require minimal training, and deliver immediate visible results. Behavioral economics research on Chinese smallholder pesticide use indicates that risk aversion and loss aversion substantially overweight the probability of immediate pest damage relative to long-term resistance development or environmental externalities (Yu et al., 2024), explaining the persistent 70%+ chemical dependency despite the availability of alternatives (**Table 8**).

**Table 8:** Technology deployment index in China’s plum industry.

Technology	Availability (%)	Deployment (%)	Gap level
Chemical control	>70	>70	Low
Biological control	~70	<10	Severe
Precision irrigation	~85	~25	High
IoT monitoring	~80	<5	Severe
Molecular breeding	~75	~15	High
Cold-chain logistics	~80	~20	High

#### 5.1.4 Storage and preservation

As climacteric fruits, plums face postharvest challenges: rapid softening, short shelf life, and high loss rates (Guo et al., 2015). Inadequate cold chain infrastructure worsens these issues, causing quality deterioration, overuse of preservatives, and price volatility (Guerra and Casquero, 2008). Key technological needs to address this include: 1) novel preservatives and modified atmosphere packaging (MAP) to extend storage life (Luo et al., 2018); 2) optimizing cold chain logistics, including formulating appropriate pre-cooling protocols and implementing intelligent temperature control systems (Yao, 2024; Yu et al., 2011); and 3) in-depth physiological research into plum deterioration mechanisms (*e.g*., mold growth, softening, browning) to develop targeted preservation strategies (Yu et al., 2011).

Identified bottlenecks: The industry has inadequate understanding of long-term storage quality regulation—requiring precise control of temperature, humidity, and gas composition. It lacks plum-specific packaging materials and bio-preservatives (*e.g*., chitosan-cinnamon essential oil coatings). Suboptimal postharvest protocols persist: pre-cooling delays, inefficient logistics, and poor-quality control. These limitations, combined with insufficient cold-chain penetration (<25% of domestic plums receive pre-cooling), result in high postharvest losses and constrain market access.

#### 5.1.5 Processing

Plum processing encompasses products including juice, dried fruits, preserves, vinegar, and wine, yet faces technical bottlenecks: unstable juice color, suboptimal dried fruit flavor, and lack of specialized starter cultures (Li et al., 2022). These issues hamper market expansion despite processing’s substantial potential: alleviating seasonal glut and reducing postharvest losses; creating high-value products (*e.g*., preserves, NFC juice) and stabilizing farmer incomes via contract farming; diversifying market offerings and enabling regional brand development; and driving upstream variety improvement and standardized cultivation (Wang et al., 2018a). However, deep processing remains underdeveloped, with most products still in primary processing and unable to meet consumer demand for healthy, functional foods (Li et al., 2022).

Identified bottlenecks: Processing limitations include unstable juice color, suboptimal dried fruit flavor, and lack of specialized microbial strains for vinegar and wine production. Deep processing remains underdeveloped, with most products still in primary processing and unable to meet consumer demand for healthy, functional foods. These constraints restrict value addition and limit the industry’s ability to capture higher-value market segments.

**Figure 5:**
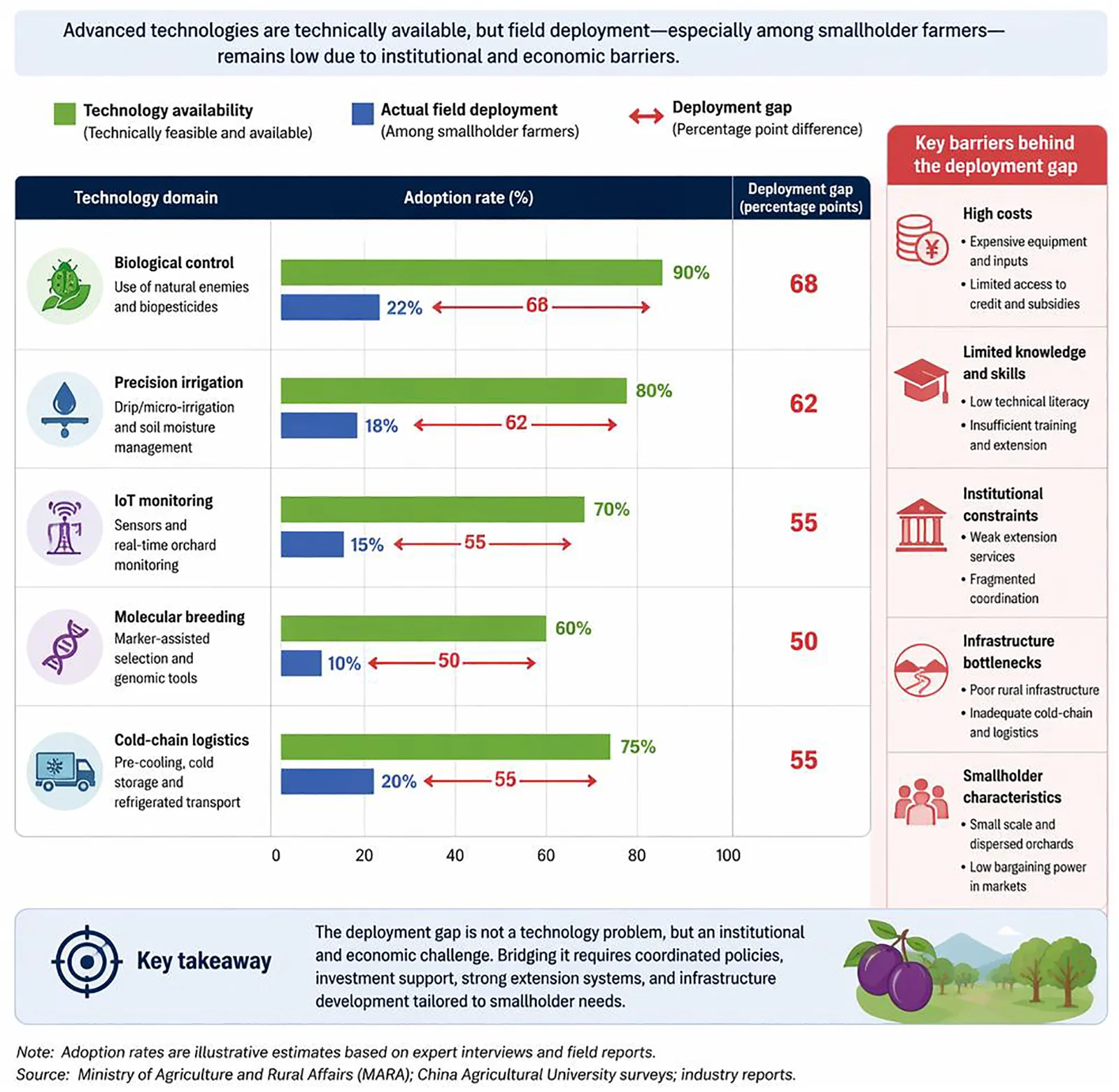
Technology deployment gap in China’s plum industry. This image visualizes the discrepancy between technology availability and actual field deployment across key domains such as biological control, precision irrigation, IoT monitoring, molecular breeding, and cold-chain logistics. It demonstrates a systemic “deployment gap,” where technologies are technically available but poorly adopted, particularly among smallholder farmers. The image underscores institutional and economic barriers rather than technological limitations.

**Figure 6:**
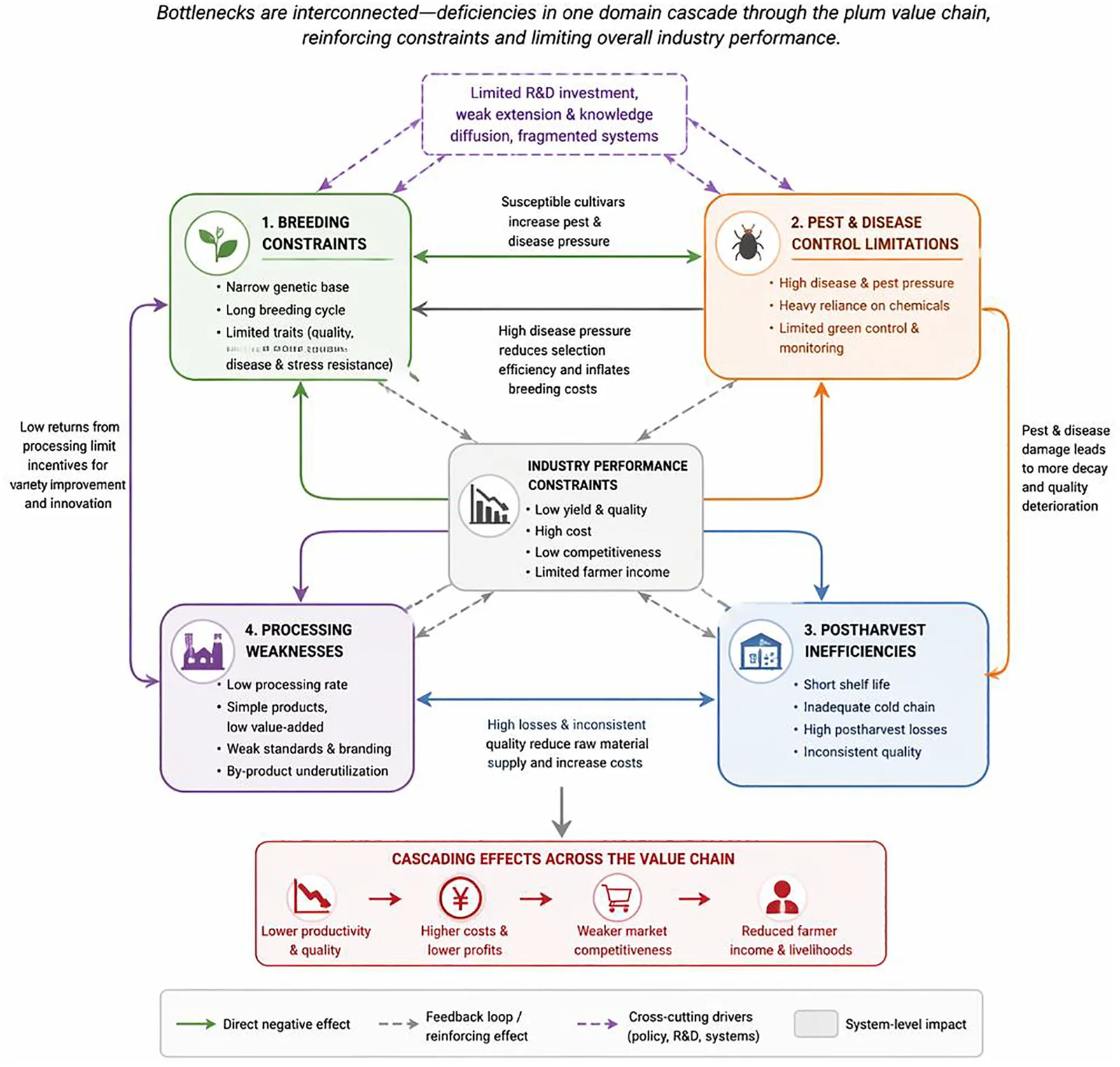
Interdependencies among technical bottlenecks. This image presents a systems-level diagram illustrating the interconnections among major bottlenecks in China’s plum industry, including breeding constraints, pest control limitations, postharvest inefficiencies, and processing weaknesses. It emphasizes feedback loops and cascading effects, showing how deficiencies in one domain (*e.g*., breeding) propagate through the entire value chain.

The cost comparison between biological and chemical control is context-dependent over time. Biological control using Trichogramma wasps for Grapholita funebrana costs ¥300–500 per mu per season (2–4 releases of 100,000–200,000 wasps at ¥0.5 per 1,000 individuals), with efficacy of 60–80% reduction in fruit damage when properly applied (Sigsgaard et al., 2017; Zhang et al., 2021). Chemical control using standard organophosphate or synthetic pyrethroid programs costs ¥200–400 per mu per season (pesticide cost ¥100–200, labor ¥80–150), with initial efficacy of 70–85% that declines with resistance development (Wang et al., 2009; Zhu and Liang, 2024). Year 1: chemical control appears marginally cheaper (¥200–400 vs. ¥300–500). Years 2–5: biological control becomes cost-equivalent or superior when factoring in: (i) EU MRL compliance costs avoided for export-oriented orchards; (ii) reduced environmental and health externalities; and (iii) declining chemical efficacy due to resistance management requiring rotation chemistries. The economic advantage of biological control is therefore realized primarily over medium-term cycles (3–5 years) rather than in the first year of adoption (**Figures 5**, **6**).

## 6 Leading research institutions in plum industry science and technology

Multiple agricultural research institutions in China are advancing the plum industry through diverse research focuses and practical applications (Liu et al., 2019).

### 6.1 Germplasm collection and preservation

This is a core research direction, with long-term efforts from key institutions. For example, the Xi’an Plum and Apricot Research Institute has dedicated decades to germplasm collection, amassing over 100 plum germplasm accessions by 2024—providing a foundational gene pool for varietal breeding (Du et al., 2023). Additionally, local characteristic varieties such as Guizhou’s ‘Suli’ and Zhejiang’s 2,500-year-old ‘Zuili’ are under systematic conservation, laying a solid base for future breeding (Zhang, 2015; Xiao et al., 2014).

### 6.2 Breeding and quality optimization

Institutions focus on developing high-quality varieties via integrated techniques. Xi’an Fengyuan Fruit Industry Technology Co., Ltd. has bred new varieties like ‘Xishuo’ and ‘Xijia’ through combining traditional hybridization and molecular biology; these varieties have higher sugar content than imported ones, with ‘Xishuo’ maintaining ~18.9% stable sugar content and ‘Xijia’ exceeding 20% (Xi'an News Network, 2024). In Sichuan, ‘Bingtangxin Li’ and ‘Fengtang Li’ have seen taste and texture improvements, with ‘Bingtangxin Li’ winning gold awards at national agricultural expos. Guangdong has achieved genetic enhancement of ‘Huangjin Naili’, developing clingstone and high-sugar traits to boost commercial value; Zhejiang has applied bagging technology to ‘Huangjin Naili’ to improve fruit appearance and storage performance.

### 6.3 Regional cultivation technique refinement

Techniques are optimized to match local ecological conditions. In Guizhou, mountain-adapted measures (terracing, drainage systems, soil amendment) and precision protocols (fertilization, pruning) have been developed to enhance plum cultivation (Zhao et al., 2024). Yunnan has established standardized cultivation for ‘Banbianhong’ plum, integrating branch pulling, water-fertilizer management, IoT traceability, and cold storage—significantly raising yield and economic benefits. In 2023, ‘Banbianhong’ plum production reached 70,000 tons (output value: 500 million yuan) (Xinhua Net, 2024b). Zhejiang has improved cultivation to address the thin-skin vulnerability of ‘Zuili’; Guizhou’s Zhenning and Jinsha counties have promoted green pest management, achieving per-mu revenues of 20,000–30,000 yuan.

### 6.4 Postharvest and processing advancements

Notable progress has been made in extending storage life and deep processing. The Liaoning Academy of Agricultural Sciences developed DENBA+ technology, which extends the storage period of ‘Dahongpao’ plums by 60 days—enhancing fruit quality and farmers’ economic returns (Yingkou Municipal People's Government, 2024). The Xinjiang branch of the National-Local Joint Engineering Research Center focuses on postharvest loss reduction, deep processing, and by-product utilization of characteristic fruits; it has also developed technical standards and promoted green circular economy models, supported by professional teams and advanced equipment (Du et al., 2023).

### 6.5 Cultural brand development and collaborative support

Beyond technology, cultural branding is gaining traction. Zhejiang leverages traditional consumption scenarios to build plum brands—for instance, promoting the vitamin-rich ‘Taoxingli’ as a specialty gift fruit to enhance market appeal (Xu et al., 2025). These efforts are backed by extensive collaborations: international partnerships with multiple countries/regions, and domestic support across research institutions. Funding comes from national key R&D programs, germplasm conservation projects, and international cooperation. Academic achievements include provincial/national awards, monographs, and publications in high-level journals.

Despite these institutional strengths, a critical coordination gap persists: no national-level plum industry coordination body exists to integrate germplasm conservation, breeding, extension, processing standards, and international market representation. In contrast, Chile’s Federación de Productores de Ciruela (FEPRUC) and the USDA National Clonal Germplasm Repository system provide centralized coordination that China currently lacks. Establishing a National Plum Industry Coordination Office under the Ministry of Agriculture and Rural Affairs, with dedicated funding for germplasm conservation, international market intelligence, and standard-setting, would address this governance gap and enable the strategic priorities outlined in Section 7.

## 7 Future research directions and strategic roadmap

Building upon the diagnostic framework established in Sections 2–6, we propose a three-tier strategic roadmap that integrates breeding, cultivation, processing, marketing, sustainability, and policy across distinct time horizons. This roadmap is grounded in empirical evidence from China’s own pilot programs (*e.g*., Liangping District smart agriculture, Yunnan ‘Banbianhong’ contract farming) and international best practices (Chilean GAP-certified value chains, EU genomic breeding consortia, Dutch automated biological control production) (Fang et al., 2020; Han et al., 2025; Sottile et al., 2022). Each tier includes explicit quantitative targets, responsible institutional actors, and identified funding mechanisms, distinguishing this roadmap from prior aspirational frameworks by its implementation specificity.

Short-Term Priorities (1–5 Years):

1. Varietal replacement acceleration: Deploy existing improved varieties (‘Guomei’, ‘Guofeng’, ‘Bingtangxin Li’) to replace low-quality local cultivars on 10% of low-output orchards annually, targeting 50% replacement by year 5. Responsible agencies: provincial agricultural departments and the National plum Germplasm Repository. Funding: Central No. 1 Document agricultural modernization grants.
2. Cold chain infrastructure rollout: Achieve 60% cold-chain penetration for domestic plum distribution by 2028; establish pre-cooling stations at 20 key production hubs in Sichuan, Chongqing, Yunnan, and Guizhou. Funding model: Public–private partnership with cold-chain logistics firms.
3. Phytosanitary compliance certification: Achieve GAP certification for 30% of export-oriented orchards by 2028, directly addressing EU MRL barriers. Link to export insurance premium reductions as incentive.
4. Extension system digitization: Integrate mobile advisory platforms and AI-based diagnostic tools into existing agricultural extension networks, reaching 500,000 smallholder users by 2027.

Medium-Term Priorities (5–10 Years):

1. Genomic breeding: Construct a 500-individual genomic selection population using SNP arrays derived from the P. salicina reference genome; release 15–20 new varieties incorporating RNAi-based PPV resistance and extended shelf-life alleles.
2. Green plant protection scale-up: Achieve 30% biological/biopesticide adoption across all plum-growing regions, reducing chemical pesticide use intensity by 50% from 2023 baseline.
3. Deep processing clusters: Establish 5–8 regional processing facilities integrated with contract-farming supply chains, targeting NFC juice, polyphenol extracts, and functional dried fruit products.
4. Market diversification: Achieve export volumes exceeding 150,000 tons/year by 2035, with >20% directed toward non-Southeast Asia markets (EU, Middle East, North America) through phytosanitary compliance and brand development.

Long-Term Innovation Goals (10+ Years):

1. Autonomous smart orchard ecosystem: Fully autonomous orchard monitoring, IoT-based pest management, and AI-driven quality grading, reducing labor costs by 60% relative to 2023 baseline.
2. Carbon-neutral plum production: Low-carbon orchard management and zero-waste circular economy models (pits-to-bioactive-compounds).
3. Global germplasm leadership: Establish China as the world’s leading Prunus germplasm repository with internationally recognized DUS testing capacity and active ITPGRFA exchange.
4. National coordination legislation: Enact a National Plum Industry Promotion Law establishing a national coordination body with standardized industry standards and international market representation.

### 7.1 Implementation milestones and phased targets

Breeding innovation: Developing multi-resistant varieties against PPV (plum pox virus), bacterial shot hole, and brown rot requires integrating genomic selection and gene editing. For example, USDA research (USDA-ARS, 2022) has successfully engineered PPV-resistant plums using RNA interference (RNAi), while Spanish studies (IRTA, 2021) identified the MYB10 gene responsible for anthocyanin accumulation, enabling targeted breeding for stable juice color in processing varieties (Scorza et al., 2013; Fiol et al., 2021). Additionally, cold-tolerant cultivars adapted to saline-alkali soils could be developed by introgression traits from wild species like *Prunus americana*.

Plant protection system: Building national pest/disease databases and AI monitoring platforms is essential. Recent applications of AI in Chongqing’s Shilong Town (People's Government of Chongqing, Banan District, 2025) demonstrate real-time pest detection and climate-driven management protocols. RNAi-based pesticides, as tested in transgenic plum rootstocks, offer targeted control of PPV, while biological agents (*e.g*., Bacillus spp.) and physical controls (*e.g*., UV-C treatment) show promise against bacterial shot hole (Leonov and Bulgakov, 2020).

Deep processing innovation: Non-thermal technologies like ultra-high pressure (UHP) and freeze-drying retain polyphenols and anthocyanins in processed products (García-Parra et al., 2014; Gouraj, 2025). Fujian’s research on Furong plums developed polyphenol-rich extracts and low-salt preservation techniques (Li et al., 2016). Zero-waste production, such as utilizing pits for bioactive compounds, aligns with circular economy goals (Savic and Savic Gajic, 2022).

Smart agriculture: Mountain-adapted machinery, including automated track transporters in Yunnan’s Suijiang County (People.cn, 2024) and lightweight fruit-detection systems (Zhang et al., 2025a), enhances harvesting efficiency. Big data models integrating soil-meteorology-pest data, as tested in Banan District, enable precision fertilization and irrigation, reducing resource waste.

The analytical evidence synthesized in this review points to a critical inflection point for China’s plum industry: the transition from a quantity-dominant production model to a quality-led, internally competitive, and internationally integrated value chain is technically feasible but requires coordinated action across six interdependent intervention domains. The five structural determinants of trade asymmetry (Section 2.3.3), the Technology Deployment Index revealing severe gaps in precisely the technologies critical for export competitiveness (Section 5), and the international benchmarking data showing China’s position at the lower end of value-chain performance (Section 4.2) collectively demonstrate that the industry’s challenges are systemic rather than isolated. Addressing them requires not merely incremental technical improvements but a strategic reorientation of institutional priorities, funding allocations, and extension delivery models—precisely the integrated approach embodied in the three-tier roadmap proposed in Section 7.

## 8 Conclusion

As a traditional advantageous agricultural sector with a globally leading production scale, China’s plum industry stands at the intersection of opportunities and challenges in the new development stage. Its strategic value extends beyond being a key component of characteristic agriculture, it also serves as a critical driver for advancing rural revitalization, optimizing agricultural industrial structures, and safeguarding national horticultural biodiversity. Against the backdrop of national policy support (*e.g*., the “14th Five-Year Plan” for seed industry development, the Central No. 1 Document’s emphasis on rural industries) and upgrading market demands (for premium, functional, and standardized plum products), the industry’s transformation and upgrading can no longer rely solely on scale expansion; instead, it must pivot toward full-value-chain technological innovation to break through existing bottlenecks.

Current constraints facing the industry are systemic and multi-dimensional: the narrow genetic base of plum germplasm limits breakthroughs in key traits (*e.g*., storage resistance, processing adaptability, stress tolerance); inadequate green plant protection systems (scarce resistant varieties, over-reliance on chemical pesticides, immature smart monitoring) hinder sustainable production; the lag in deep processing technologies (unstable product quality, limited functional product development) restricts value elevation; and the “high import, low export” trade imbalance (exports accounting for only 1–2% of total output) reflects a gap in international competitiveness compared to superior varieties from Chile, the U.S., and other countries. These challenges, however, also align with the core research directions identified in this review—addressing them requires targeted efforts in germplasm resource enrichment (systematic collection of wild relatives and local varieties), precision breeding (integrating genomic selection and gene editing for multi-resistant, high-quality varieties), green production (promoting AI-driven pest monitoring and biological control), and value-added processing (applying non-thermal technologies and zero-waste utilization).

Looking ahead, the sustainable development of China’s plum industry hinges on three pillars: interdisciplinary collaboration, institutional support, and international alignment will accelerate the translation of basic research (*e.g*., physiological mechanisms of fruit quality formation, stress resistance genetics) into practical technologies (*e.g*., precision cultivation protocols, smart cold chain systems). Strengthened institutional support—including increased funding for germplasm conservation, the establishment of national-level industry standards, and the promotion of “variety + technology + brand” integrated models—will provide a stable framework for industrial upgrading. Alignment with international standards (*e.g*., EU regulations for pesticide residues, global quality criteria for fresh and processed plums) will help expand high-end market access, reversing the trade deficit and enhancing the industry’s global voice.

Ultimately, China’s plum industry is poised to transition from a “quantity-dominant” model to a “quality-led” one. By integrating traditional cultivation wisdom (*e.g*., the 2,500-year-old cultivation history of Zhejiang’s ‘Zuili’) with modern science and technology (*e.g*., gene editing, IoT-based orchard management), the industry will not only achieve higher agricultural efficiency and sustained farmer income growth but also contribute significantly to the realization of rural revitalization and the building of a strong agricultural nation. In this process, it will also set a paradigm for the transformation of other traditional horticultural industries—demonstrating how localized advantages can be upgraded into global competitiveness through innovation.
